# The relationship between fundamental motor skills and executive function in children: a stratified analysis by age and sex

**DOI:** 10.3389/fpsyg.2025.1669915

**Published:** 2025-09-05

**Authors:** Chuifeng Kong, Yujun Cai, Kai Li, Zisu Wang, Xili Wen, Xinmiao Zhang

**Affiliations:** ^1^School of Physical Education, Shanghai University of Sport, Shanghai, China; ^2^College of Sports Industry and Leisure, Nanjing Sport Institute, Nanjing, China; ^3^Department of Sports Science, Zhejiang University, Hangzhou, China

**Keywords:** children, fundamental motor skills, executive function, age, gender

## Abstract

**Objective:**

This study aimed to systematically examine the relationship between fundamental motor skills (FMS) and executive function (EF) in Chinese children aged 3 to 10 years. The study specifically focused on age and sex differences, as well as the associations between subcomponents of FMS and EF, to provide empirical evidence for research and intervention strategies targeting motor-cognitive integration in child development.

**Methods:**

A cross-sectional study design was employed, utilizing multi-stage stratified cluster sampling to recruit 2,179 children aged 3 to 10 years from eight cities across four eastern provinces in China (Jiangsu, Zhejiang, Anhui, and Shandong). FMS were assessed using the Test of Gross Motor Development-Third Edition (TGMD-3), which evaluates two major domains: locomotor skills and object control skills. EF was measured using the Childhood Executive Functioning Inventory (CHEXI), a parent-report scale that includes four subcomponents: working memory, inhibition, regulating ability, and planning ability. Statistical analyses included correlation analysis, and hierarchical regression modeling to examine both overall and stratified associations between FMS and EF by age and sex.

**Results:**

A significant negative correlation was found between fundamental motor skills and executive function, indicating that higher motor skill levels were associated with better EF performance. Age-stratified analyses revealed that this association was significantly stronger in the 3–5 years group compared to the 6–10 years group, demonstrating clear age specificity. Sex-stratified results showed that for boys, the association between object control skills and EF, as well as between inhibition and FMS, was stronger. In contrast, girls exhibited significant associations between locomotor skills and EF, inhibition and FMS, and regulating ability and FMS.

**Conclusion:**

This study confirms significant associations between fundamental motor skills and executive function in Chinese children, revealing distinct age-specific and sex-dependent patterns. The findings emphasize the critical importance of implementing targeted motor interventions during key developmental periods, providing both theoretical support and practical guidance for promoting the coordinated development of motor competence and higher-order cognitive functions in children.

## 1 Introduction

Childhood constitutes a critical period for the development of motor competence (MC) (Escolano-Pérez et al., 2022; Albuquerque et al., 2022; Zhou and Tolmie, 2024; Van Fels et al., 2019). During this phase, children’s MC is primarily reflected in their proficiency in fundamental motor skills (FMS) (Cattuzzo et al., 2016; Utesch et al., 2019), which play a pivotal role in facilitating language, cognitive, and social development (Malambo et al., 2022; Rosenbaum et al., 2001; Bar-Haim and Bart, 2006; Iverson, 2010; Gandotra et al., 2023; Macdonald et al., 2020). FMS are culturally acquired foundational motor patterns that form the neurobehavioral basis for engaging in complex physical activities and sport-specific skills (Barnett et al., 2016). Most research globally categorizes FMS into three main domains: locomotor skills, object control skills, and stability skills (Xin et al., 2019; Gabbard, 2021; Diao, 2018; Rudd et al., 2015). A large body of evidence confirms that FMS not only provide the foundation for more complex motor tasks but also directly influence lifelong participation in physical activities and the adoption of a healthy lifestyle (Stodden et al., 2008; Escolano-Pérez et al., 2022; Zhou and Tolmie, 2024; Gu et al., 2021). The development of FMS is influenced by biological factors such as age and sex (Gandotra et al., 2023; Navarro-Patón et al., 2021b; Chichinina et al., 2025), and may also involve key elements of executive function (EF), which is considered a core foundation of overall child development (Garon et al., 2008). Since Piaget’s theory of the motor-cognition linkage, the Northern Finland Birth Cohort study (1966) has demonstrated that early walking ability significantly predicts executive function 35 years later (Murray et al., 2006; Ridler et al., 2006). As research in this area has advanced into the 21st century, scholars have increasingly focused on investigating the reciprocal interactions between FMS and EF during early childhood (Diamond, 2000). Understanding these interactions holds significant theoretical and practical value for developing evidence-based interventions and optimizing child development outcomes.

Executive function (EF) serves as a core cognitive mechanism in child development (Van Fels et al., 2019), playing a determinant role in key developmental indicators such as psychological well-being, academic achievement, and social adaptation (Albuquerque et al., 2022). Research has shown that during childhood, EF significantly predicts the quality of transition to formal education by supporting the development of behavioral competencies, preschool skill acquisition, and social relationship formation (Willoughby and Hudson, 2023). EF refers to cognitive processes involved in purposeful, goal-directed behavior (Stuss, 1992), achieved through the coordination of fundamental cognitive domains, including language, attention, perception, and motor functions (Neisser, 2014), These processes enable problem-solving, reasoning, planning, and behavioral regulation (Isquith et al., 2005). There is broad consensus that EF encompasses core components such as inhibitory control, working memory, cognitive flexibility, attentional monitoring, planning initiation, and self-regulation (Miyake and Friedman, 2012; Goldstein, 2014). A among these, Miyake et al. (2000) tripartite model, which includes working memory, inhibitory control, and cognitive flexibility, is widely accepted. Recently, researchers have increasingly highlighted planning capacity as a vital component of EF (Diamond, 2013; Anderson and Reidy, 2012). The Childhood Executive Functioning Inventory (CHEXI), developed based on Barkley’s (1997) hybrid model (Thorell and Nyberg, 2008; Camerota et al., 2018; Moura et al., 2025; Wei et al., 2018), assesses EF through four factors: working memory, planning ability, regulation, and inhibition.

Existing research has established significant age-dependent associations between FMS and EF (Albuquerque et al., 2022; Libertus and Hauf, 2017; Luz et al., 2015; Cook et al., 2019; Liu et al., 2022), with particularly pronounced linkages in younger children that progressively attenuate with advancing age (Albuquerque et al., 2022). This attenuation trajectory may arise from two key neurodevelopmental mechanisms. First, core EF-related brain regions—especially the prefrontal cortex—undergo considerably prolonged developmental cycles compared to motor cortices (Davis et al., 2011), leading to a relative decline in the direct contribution of EF to motor performance after the preschool years (Stuhr et al., 2020). Second, the synergistic relationships among EF subcomponents, such as working memory and inhibitory control, follow a nonlinear developmental trajectory (Brocki and Bohlin, 2004), with significantly stronger coupling of working memory and inhibition observed in 6- to 8-year-olds compared to 9- to 11-year-olds (Spedden et al., 2017). Separately, critical motor milestones in infancy (such as independent walking) significantly predict EF performance in adulthood (Ridler et al., 2006), suggesting a potential neurodevelopmental covariation between early motor experiences and later cognitive abilities. Overall, current research on the relationship between motor and cognitive development predominantly focuses on school-aged children, limiting the generalizability of findings to preschool populations (Gandotra et al., 2023). To fully elucidate the age-specific nature of this association, it is essential to examine a broader age range. Accordingly, this study investigates children aged 3 to 10 years, encompassing both preschool and school-age developmental phases, with the goal of analyzing the age-related characteristics of the FMS-EF association.

Furthermore, existing research suggests that sex-based differences exist in the association patterns between FMS and EF (Mileva-Seitz et al., 2015; Yang et al., 2022). Typically, boys exhibit stronger overall FMS-EF linkages than girls (Yang et al., 2022). At the subcomponent level, boys show significant associations between object control skills and EF, whereas girls demonstrate stronger locomotor-EF linkages (Escolano-Pérez et al., 2022; Hirata et al., 2018; Ke et al., 2020; Smits-Engelsman et al., 2023; Navarro-Patón et al., 2021a; Mecías-Calvo et al., 2021). Notably, self-regulatory ability within EF is exclusively associated with girls’ FMS proficiency (Mileva-Seitz et al., 2015). Overall, current evidence on sex-differentiated FMS-EF associations is limited, particularly regarding subcomponent-specific relationships. Therefore, sex-stratified analysis of children aged 3–10 years is a secondary research priority of this study, aimed at clarifying sex-dependent characteristics of FMS-EF linkages.

This study systematically examines the age- and sex-dependent associations between FMS and EF in Chinese children aged 3–10 years, using a cross-sectional design. This is the first such investigation conducted in this population. Using standardized assessments, we evaluated locomotor and object control skills (FMS subdomains) (Xin et al., 2019; Gabbard, 2021; Diao, 2018; Rudd et al., 2015) alongside core EF components: working memory, inhibitory control, cognitive flexibility, and planning capacity (Miyake and Friedman, 2012; Goldstein, 2014). Guided by sensitive period theory in motor development and cognitive developmental stage theory, participants were stratified into two theoretically-informed groups: 3-5-year-olds (sensorimotor exploration and self-identity construction phase) and 6-10-year-olds (cognitive system transformation and motor specialization phase). Based on this theoretical framework, we propose the following hypotheses:

1. Significant bidirectional associations exist between FMS and EF in 3-10-year-olds, where FMS potentially influences EF development, and reciprocally, EF potentially influences FMS development.
2. FMS-EF association patterns demonstrate age-specific characteristics across developmental phases.
3. FMS-EF association patterns exhibit sex-dependent characteristics.

## 2 Methods

### 2.1 Study design and participants

This study enrolled 2,560 Chinese children aged 3–10 years using a multistage stratified cluster sampling method.

Stage 1: Geographical and Socioeconomic Stratification. Four eastern provinces (Jiangsu, Zhejiang, Anhui, Shandong) were selected based on regional economic distribution. Within each province, one high-income and one low-income city were identified using per capita disposable income data, resulting in eight cities: Changzhou, Yancheng, Hangzhou, Taizhou, Wuhu, Anqing, Jinan, and Liaocheng.
Stage 2: Educational Institution Stratification. In each city, one primary school and one kindergarten were selected from both urban and suburban districts, yielding a total of 32 institutions.
Stage 3: Classroom-level Cluster Sampling. From each selected classroom, 20–25 children were randomly recruited, resulting in 2,560 participants.

The minimum required sample size was determined using G*Power software (version 3.1.9.7) (Faul et al., 2009). Based on parameters for a two-tailed correlation test (effect size f^2^ = 0.02, *α* = 0.05, statistical power [1-*β*] = 0.95, and a maximum of 5 predictor variables for hierarchical regression), the calculated minimum sample size was 776 participants. Therefore, the final sample size was adjusted to account for the design effect associated with cluster sampling and potential missing data.

Ethical approval was granted by the Shanghai University of Sport Institutional Review Board (Approval No: 102772021RT072). Written informed consent was obtained from all legal guardians, and strict adherence to voluntary participation principles was maintained throughout the study.

### 2.2 Procedures

All participants underwent assessments for both fundamental motor skills (FMS) and executive function (EF). FMS were evaluated using the Test of Gross Motor Development, Third Edition (TGMD-3). During the assessment, a strategic arrangement of six to eight high-definition cameras was set up within the testing area to capture the movements of all 2,560 children. The testing was conducted by a team of four to six graduate students specializing in physical education. These individuals were responsible for participant registration, test administration, operation of the multiple camera units, and providing standardized demonstrations of the required motor skills. Subsequently, three graduate students, who had received specific training in TGMD-3 administration, independently scored the recorded performances based on standardized scoring criteria for locomotor skills (e.g., running and hopping) and object control skills (e.g., overhand throwing and two-hand catching).

Executive function was measured using paper-based questionnaires. These questionnaires were distributed and completed immediately after the motor skills assessment. A total of 2,230 completed questionnaires were collected, yielding a response rate of 87.1%. After performing quality control checks for completeness and logical consistency, 51 invalid questionnaires were excluded. This resulted in 2,179 valid questionnaires, representing an effective response rate of 97.7%. Ultimately, complete data sets, encompassing both FMS testing and EF assessment, were obtained for 2,179 children (1,079 boys and 1,100 girls), ensuring the integrity of the data.

### 2.3 Measures

#### 2.3.1 Anthropometry

Demographic information was collected for each participating child, including sex, age, ethnicity, height, weight, and body mass index (BMI).

#### 2.3.2 Fundamental motor skills

Fundamental motor skills were assessed using the Test of Gross Motor Development, Third Edition (TGMD-3). This instrument is designed for evaluating children aged 3 to 10 years and consists of two subtests: locomotor skills and object control skills, which together encompass a total of thirteen test items. Each test item includes three to five performance criteria. One point is awarded for each criterion successfully met during a trial, and zero points are awarded if a criterion is not met. Each item is administered twice, with a maximum possible score of 100 points. The TGMD-3 enables comparisons both between individuals and across groups and is suitable for use in research and educational settings. The instrument has demonstrated satisfactory applicability for Chinese children aged 3 to 12 years (Li et al., 2022). Internal consistency coefficients (Cronbach’s alpha) for the fundamental motor skill scores within each age group ranged from 0.808 to 0.902, with coefficients reaching 0.95 for both male and female subgroups, indicating good internal reliability.

Furthermore, to evaluate inter-rater reliability specifically for this study (as presented in Table 1), three independent raters scored video recordings of a random sample of fifty children. The resulting ICCs, calculated from this study’s data, showed high inter-rater agreement, with all intraclass correlation coefficients (ICCs) exhibiting statistical significance (*p* < 0.001). Specifically, ICCs for the locomotor skills subtest total scores ranged from 0.822 to 0.914, ICCs for the object control skills subtest total scores ranged from 0.886 to 0.940, and ICCs for the overall TGMD-3 total scores ranged from 0.931 to 0.953. These findings support the TGMD-3 as a valid and reliable assessment tool for evaluating fundamental motor skill development in Chinese children (Li et al., 2022).

**Table 1:** Inter-rater reliability coefficients for TGMD-3 scoring.

Motor skill items	T1 & T2	T1 & T3	T2 & T3
Run	0.593^**^	0.705^**^	0.588^**^
Hop	0.793^**^	0.801^**^	0.730^**^
Horizontal jump	0.583^**^	0.626^**^	0.815^**^
Slide	0.435^*^	0.679^**^	0.885^**^
Gallop	0.667^**^	0.601^**^	0.562^**^
Skip	0.899^**^	0.813^**^	0.892^**^
Locomotor subtest raw score	0.822^**^	0.866^**^	0.914^**^
Overhand throw	0.897^**^	0.918^**^	0.926^**^
Underhand roll	0.824^**^	0.832^**^	0.844^**^
Stationary dribble	0.593^**^	0.449^*^	0.460^*^
Catch	0.749^**^	0.798^**^	0.856^**^
Kick	0.598^**^	0.730^**^	0.601^**^
Striking a stationary ball	0.868^**^	0.544^**^	0.464^*^
Forehand strike	0.579^**^	0.731^**^	0.791^**^
Object control skills subtest raw score	0.886^**^	0.940^**^	0.926^**^

**p < 0.01, *p < 0.05. T1 = rater 1; T2 = rater 2; T3 = rater 3.

#### 2.3.3 Executive function

Executive function was assessed using the Childhood Executive Functioning Inventory (CHEXI) (Wei et al., 2018). This instrument is designed for use with populations ranging from preschool to adolescence and is completed by parents or teachers. The CHEXI systematically evaluates four core components of executive function through a total of 24 items, organized into subscales measuring working memory, planning ability, regulatory ability, and inhibitory control. Responses are recorded using a five-point Likert scale (1 = completely untrue, 5 = completely true). Subscale scores and a total score are calculated separately. The instrument employs reverse scoring, with higher scores indicating poorer executive function, and therefore, lower scores represent better executive function.

Among Chinese children, the CHEXI has demonstrated satisfactory reliability, with Cronbach’s *α* coefficients for the working memory, regulatory ability, and inhibitory control subscales ranging from 0.71 to 0.89. These psychometric properties confirm its suitability for early childhood development research and clinical assessment of executive function (Tsai et al., 2020; Wei et al., 2018).

#### 2.3.4 Statistical analysis

Data management and statistical analyses were performed using SPSS version 26.0 (IBM, Chicago, IL, United States), with statistical significance set at *p* < 0.05. Descriptive statistics were used to analyze the total scores of FMS, EF, as well as their subcomponents, along with variables such as sex, age, body mass index (BMI), geographical region, and ethnicity. Pearson correlation analysis was employed to assess the relationship between FMS and EF. Hierarchical linear regression modeling was used to explore the bidirectional predictive relationships between FMS and EF. Specifically, this analysis assessed both the potential predictive relationship of FMS on EF and the reciprocal predictive relationship of EF on FMS. Further analyses examined variations in these relationships across different sex and age groups. In the hierarchical regression analysis, Model 1 included only control variables (BMI, region, and sex) to evaluate the influence of these covariates on the dependent variables. Model 2 added the core independent variable (either FMS or EF) to examine its independent contribution to the dependent variables after controlling for covariates.

## 3 Results

### 3.1 Sample characteristics

Sample characteristics are presented in Table 2. Children demonstrated a mean FMS total score of 68.74 ± 13.91, with locomotor skills at 35.11 ± 7.07 and object control skills at 33.64 ± 8.50. The mean EF total score was 58.79 ± 15.04, comprising working memory (22.08 ± 6.10), planning ability (11.39 ± 2.97), regulating ability (12.03 ± 3.71), and inhibition (13.29 ± 4.19).

**Table 2:** Descriptive statistics of demographic information and independent and dependent variables.

Categorical variables	*n*	%
Sex		
Boys	1,079	49.5
Girls	1,100	50.5
Age(years)		
3–5	851	39.1
6–10	1,328	60.9
Area		
Urban	1,089	50.0
Suburban	1,090	50.0
Nationality		
Han	2,140	98.2
Minority	39	1.8

Continuous variables	Mean	SD
BMI	16.48	2.65
FMS		
Locomotor	35.11	7.07
Object control	33.64	8.50
Total	68.74	13.91
EF		
Working memory	22.08	6.10
Planning ability	11.39	2.97
Regulating ability	12.03	3.71
Inhibition	13.29	4.19
Total	58.79	15.04

FMS: Fundamental motor skills; EF: Executive function; hereafter identical.

### 3.2 Correlational analysis

**Figure 1:**
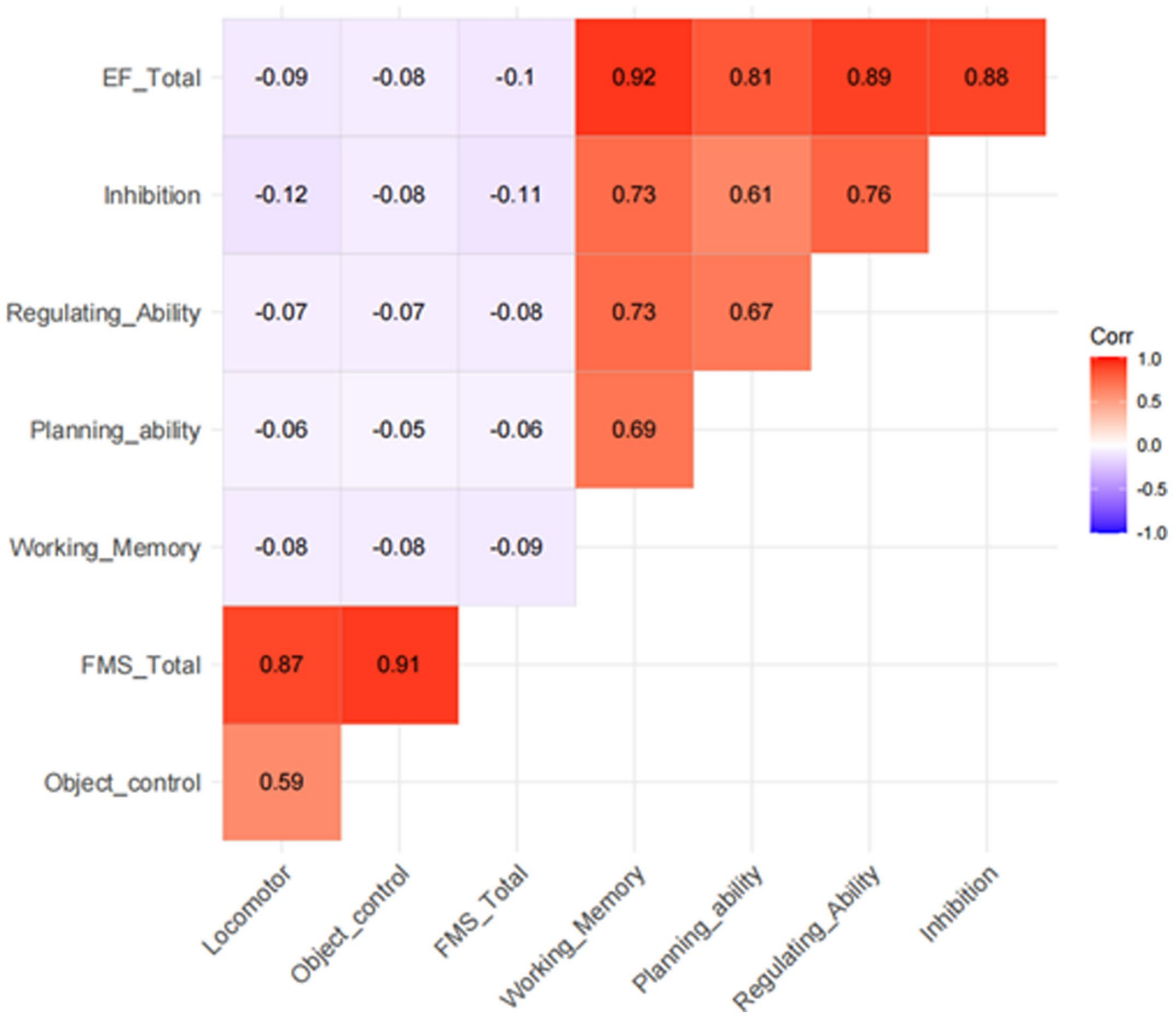
Correlation analysis between fundamental motor skills and executive function in children.

Correlation analysis results (Figure 1) revealed significant negative correlations between fundamental motor skills (total score, locomotor skills, and object control skills) and executive function (total score and all subcomponents: working memory, planning ability, regulatory ability, inhibitory ability) in 3–10-year-old children (r = −0.06 to −0.12, *p* < 0.05), indicating small effect sizes per Cohen’s criteria (r = 0.10 for small effect) (Cohen, 2013). Notably, the strongest negative correlation was observed between inhibitory ability and locomotor skills (r = −0.12, *p* < 0.01), approaching a small-to-medium effect size (r = 0.30 for medium effect). Additionally, high positive intercorrelations were found among all executive function subcomponents (r = 0.61 to 0.92, *p* < 0.01), representing large to very large effect sizes (r = 0.50 for large effect).

### 3.3 The relationship between fundamental movement skills and executive function in children: age stratification

Hierarchical regression analyses (Table 3) demonstrated that when executive function served as the dependent variable in the 3–5-year-old group, Model 1 (control variables) explained 1.9% of the variance (*F* = 5.602, *p* < 0.01). Following the addition of fundamental motor skills in Model 2, the explained variance significantly increased to 4.4% (*F* = 9.625, *p* < 0.001), with fundamental motor skills exhibiting a significant negative association (*β* = −0.157, *t* = −4.614, *p* < 0.001). In the 6–10-year-old group, Model 1 accounted for 1.3% of the variance (*F* = 5.593, *p* < 0.001). After incorporating fundamental motor skills in Model 2, the explained variance increased to 1.7% (*F* = 5.702, *p* < 0.001), and fundamental motor skills maintained a significant negative association (*β* = −0.069, *t* = −2.442, *p* < 0.05). When fundamental motor skills were designated as the dependent variable, Model 1 explained 2.0% of the variance in the 3–5-year-old group (*F* = 6.340, *p* < 0.001). After adding executive function in Model 2, the explained variance increased to 4.9% (*F* = 9.814, *p* < 0.001), with executive function showing a significant negative association (*β* = −0.157, *t* = −4.614, *p* < 0.001). For the 6–10-year-old group, Model 1 accounted for 7.7% of the variance (*F* = 17.258, *p* < 0.001). Following the inclusion of executive function in Model 2, the explained variance reached 8.1% (*F* = 29.164, *p* < 0.001), and executive function retained a significant negative association (*β* = −0.065, *t* = −2.442, *p* < 0.05).

**Table 3:** Cross-sectional association between fundamental motor skills and executive function: hierarchical regression analysis by age group.

Association direction	Predictor variables	3–5 years (*n* = 851)		6–10 years (*n* = 1,328)	
		Model 1 β	Model 2 β	Model 1 β	Model 2 β
A. Outcome: EF					
	Control variables				
	BMI	0.077*	0.067*	−0.006	−0.002
	Region (Rural = 0; Urban = 1)	0.110*	0.096*	0.052	0.046
	Gender (Female = 0; Male = 1)	−0.030	−0.045	−0.100*	−0.117*
	Core variable				
	Fundamental motor skills	—	−0.157***	—	−0.069*
	Model statistics				
	R^2^	0.019	0.044	0.013	0.017
	ΔR^2^	0.019	0.024	0.013	0.004
	F	5.602**	9.625***	5.593**	5.702***
B. Outcome: FMS					
	Control variables				
	BMI	−0.068**	−0.055	0.062**	0.062**
	Region (Rural = 0; Urban = 1)	−0.089***	−0.072**	−0.089***	−0.086***
	Gender (Female = 0; Male = 1)	−0.093***	−0.098***	−0.247***	−0.254***
	Core variable				
	Executive function	—	−0.157***	—	−0.065*
	Model statistics				
	R^2^	0.020	0.049	0.077	0.081
	ΔR^2^	0.020	0.029	0.077	0.004
	F	5.848**	9.814***	36.760***	29.164***

***p < 0.001, **p < 0.01, *p < 0.05; A: Outcome = Executive function; Core predictor = Fundamental motor skills; B: Outcome = Fundamental motor skills; Core predictor = Executive function.

Further analysis examining subcomponents (Table 4) revealed that when executive function was the dependent variable in the 3–5-year-old group, Model 1 explained 1.9% of the variance (*F* = 5.602, *p* < 0.001). After adding locomotor skills in Model 2, the explained variance significantly increased to 4.4% (*F* = 7.710, *p* < 0.001), with locomotor skills demonstrating a significant negative association (*β* = −0.106, *t* = −2.620, *p* < 0.01). In the 6–10-year-old group, Model 1 accounted for 1.3% of the variance (*F* = 5.593, *p* < 0.001). Following the inclusion of object control skills in Model 2, the explained variance increased to 1.8% (*F* = 4.830, *p* < 0.001), and object control skills showed a significant negative association (*β* = −0.076, *t* = −2.508, *p* < 0.01). When fundamental motor skills served as the dependent variable in the 3–5-year-old group, Model 1 explained 2.0% of the variance (*F* = 5.848, *p* < 0.001). After adding inhibitory ability in Model 2, the explained variance increased to 5.0% (*F* = 6.340, *p* < 0.001), with inhibitory ability exhibiting a significant negative association (*β* = −0.158, *t* = −2.747, *p* < 0.001). In the 6–10-year-old group, Model 1 accounted for 7.7% of the variance (*F* = 36.760, *p* < 0.001). Following the incorporation of working memory in Model 2, the explained variance reached 8.4% (*F* = 17.258, *p* < 0.001), and working memory demonstrated a significant negative association (*β* = −0.088, *t* = −1.961, *p* < 0.001).

**Table 4:** Cross-sectional association between subcomponents of fundamental motor skills and executive function: hierarchical regression analysis by age group.

Association direction	Predictors	3–5 years (*n* = 851)		6–10 years (*n* = 1,328)	
		Model 1 β	Model 2 β	Model 1 β	Model 2 β
A. Outcome: EF					
	Control variables				
	BMI	0.077*	0.066*	−0.006	−0.002
	Region (Rural = 0; Urban = 1)	0.110**	0.096**	0.052	0.046
	Gender (Female = 0; Male = 1)	−0.030	−0.042	−0.100***	−0.117***
	Core FMS components				
	Locomotor	-	−0.106**	-	-
	Object control	-	-	-	−0.076*
	Model statistics				
	R^2^	0.019	0.044	0.013	0.018
	ΔR^2^	0.019	0.024	0.013	0.005
	F	5.602***	7.710***	5.593***	4.830***
B. Outcome: FMS					
	Control variables				
	BMI	−0.068*	−0.055	0.062*	0.062*
	Region (Rural = 0; Urban = 1)	−0.089**	−0.072*	−0.089***	−0.086***
	Gender (Female = 0; Male = 1)	−0.093**	−0.098***	−0.247***	−0.254***
	Core EF components				
	Inhibitory ability	-	−0.158**	-	-
	Working memory	-	-	-	−0.088*
	Model statistics				
	R^2^	0.020	0.050	0.077	0.084
	ΔR^2^	0.020	0.030	0.077	0.007
	F	5.848***	6.340***	36.760***	17.258***

***p < 0.001, **p < 0.01, *p < 0.05; A: Outcome = Executive function; Core predictors = Locomotor and Object control (subcomponents of fundamental motor skills); B: Outcome = Fundamental motor skills; Core predictors = Inhibitory ability and Working memory (subcomponents of executive function).

### 3.4 Associations between fundamental motor skills and executive function in children: stratified by sex

Hierarchical regression analyses (Table 5) demonstrated that when executive function served as the dependent variable in the male group, Model 1 (control variables) explained 0.9% of the variance (*F* = 3.301, *p* < 0.05). Following the addition of fundamental motor skills in Model 2, the explained variance significantly increased to 1.6% (*F* = 4.408, *p* < 0.01), with fundamental motor skills exhibiting a significant negative predictive association (*β* = −0.123, *t* = −2.769, *p* < 0.01). In the female group, Model 1 accounted for 1.7% of the variance (*F* = 6.229, *p* < 0.001). After incorporating fundamental motor skills in Model 2, the explained variance reached 2.0% (*F* = 5.703, *p* < 0.001), and fundamental motor skills maintained a significant negative association (*β* = −0.090, *t* = −2.018, *p* < 0.01). When fundamental motor skills were designated as the dependent variable in the male group, Model 1 explained 53.4% of the variance (*F* = 411.167, *p* < 0.001). After adding executive function in Model 2, the explained variance increased to 53.8% (*F* = 312.205, *p* < 0.001), with executive function showing a significant negative association (*β* = −0.058, *t* = −2.769, *p* < 0.01). In the female group, Model 1 accounted for 55.3% of the variance (*F* = 452.584, *p* < 0.001). Following the inclusion of executive function in Model 2, the explained variance reached 55.5% (*F* = 341.408, *p* < 0.001), and executive function retained a significant negative association (*β* = −0.041, *t* = −2.018, *p* < 0.01).

**Table 5:** Cross-sectional association between fundamental motor skills and executive function: hierarchical regression analysis by sex.

Association direction	Predictors	Male (*n* = 1,079)		Female (n = 1,100)	
		Model 1 β	Model 2 β	Model 1 β	Model 2 β
A. Outcome: EF					
	Control variables				
	BMI	0.064*	0.056	0.006	0.001
	Region (Rural = 0; Urban = 1)	0.055	0.047	0.094**	0.087**
	Age (centered)	−0.070*	0.022	−0.092**	−0.024
	Core variable				
	Fundamental motor skills	-	−0.123**	-	−0.090*
	Model statistics				
	R^2^	0.009	0.016	0.017	0.020
	ΔR^2^	0.009	0.007	0.017	0.004
	F	3.301*	4.408**	6.229***	5.703***
B. Outcome: FMS					
	Control variables				
	BMI	−0.065**	−0.061**	−0.053*	−0.052*
	Region (Rural = 0; Urban = 1)	−0.068**	−0.063**	−0.081***	−0.077***
	Age (centered)	0.744***	0.740***	0.752***	0.748***
	Core variable				
	Executive functions	-	−0.058**	-	−0.041*
	Model statistics				
	R^2^	0.534	0.538	0.553	0.555
	ΔR^2^	0.534	0.003	0.553	0.002
	F	411.167***	312.205***	452.584***	341.408***

***p < 0.001, **p < 0.01, *p < 0.05; A: Outcome = Executive function; Core predictor = Fundamental motor skills; B: Outcome = Fundamental motor skills; Core predictor = Executive function.

Further subcomponent analyses (Table 6) revealed that when executive function was the dependent variable in the male group, Model 1 explained 0.9% of the variance (*F* = 3.301, *p* < 0.01). After adding object control skills in Model 2, the explained variance significantly increased to 1.7% (*F* = 4.650, *p* < 0.001), with object control skills demonstrating a significant negative association (*β* = −0.132, *t* = −2.937, *p* < 0.01). In the female group, Model 1 accounted for 1.7% of the variance (*F* = 6.229, *p* < 0.001). Following the inclusion of locomotor skills in Model 2, the explained variance reached 2.2% (*F* = 6.020, *p* < 0.001), and locomotor skills showed a significant negative association (*β* = −0.082, *t* = −2.306, *p* < 0.05). When fundamental motor skills served as the dependent variable in the male group, Model 1 explained 53.4% of the variance (*F* = 411.167, *p* < 0.001). After adding inhibitory ability in Model 2, the explained variance increased to 53.9% (*F* = 313.929, *p* < 0.001), with inhibitory ability exhibiting a significant negative association (*β* = −0.069, *t* = −3.298, *p* < 0.01). In the female group, Model 1 accounted for 55.3% of the variance (*F* = 452.584, *p* < 0.001). Following the incorporation of inhibitory ability in Model 2, the explained variance reached 55.8% (*F* = 344.978, *p* < 0.001), and inhibitory ability maintained a significant negative association (*β* = −0.066, *t* = −3.233, *p* < 0.01). When regulatory ability was further added in Model 3, the explained variance increased to 56.0% (*F* = 277.677, *p* < 0.001). The magnitude of association for inhibitory ability strengthened (*β* = −0.114, *t* = −3.689, *p* < 0.001), while regulatory ability showed a positive association (*β* = 0.064, *t* = 2.075, *p* < 0.05).

**Table 6:** Cross-sectional associations between subcomponents of fundamental motor skills and executive function: hierarchical regression analyses stratified by sex.

Association direction	Predictors	Male (*n* = 1,079)		Female (*n* = 1,100)		
		Model 1 β	Model 2 β	Model 1 β	Model 2 β	Model 3 β
A. Outcome: EF						
	Control variables					
	BMI	0.064*	0.056	0.006	−0.001	
	Region (Rural = 0; Urban = 1)	0.055	0.047	0.094**	0.091**	-
	Age (centered)	−0.070*	0.030	−0.092**	−0.046	-
	Core variables					
	Object control	-	−0.132**	-	-	-
	Locomotor	-	-	-	−0.082*	-
	Model statistics					
	R^2^	0.009	0.017	0.017	0.022	-
	ΔR^2^	0.009	0.008	0.017	0.005	-
	F	3.301*	4.650***	6.229***	6.020***	-
B. Outcome: FMS						
	Control variables					
	BMI	−0.065**	−0.061**	−0.053*	−0.051*	−0.050*
	Region (Rural = 0; Urban = 1)	−0.068**	−0.063**	−0.081***	−0.072***	−0.071***
	Age (centered)	0.744***	0.740***	0.752***	0.747***	0.748***
	Core variables					
	Inhibition	-	−0.069**	-	−0.066**	−0.114***
	Regulating ability	-	-	-	-	0.064*
	Model statistics					
	R^2^	0.534	0.539	0.553	0.558	0.559
	ΔR^2^	0.534	0.005	0.533	0.004	0.002
	F	411.167***	313.929***	452.584***	344.978***	277.677***

***p < 0.001, **p < 0.01, *p < 0.05; A: Outcome = Executive function; Core predictors = Object control and Locomotor (subcomponents of fundamental motor skills); B: Outcome = Fundamental motor skills; Core predictors = Inhibition and Regulating ability (subcomponents of executive function).

## 4 Discussion

This study revealed a significant negative association between fundamental motor skills and executive function, with higher scores in fundamental motor skills corresponding to lower scores in executive function, and conversely, lower fundamental motor skills scores being associated with higher executive function scores. This relationship demonstrated age-specific patterns, with stronger associations observed in the 3–5-year-old group compared to the 6–10-year-old group. Subcomponent analyses further revealed that among preschool-aged children, locomotor skills exhibited significant associations with executive function, while inhibitory ability was significantly associated with fundamental motor skills. Conversely, in school-aged children, object control skills demonstrated significant associations with executive function, and working memory showed significant associations with fundamental motor skills. Sex-stratified analyses indicated that the reciprocal relationship between overall fundamental motor skills and executive function was stronger in boys than in girls. Subcomponent associations also exhibited sex-specific patterns: Object control skills were significantly associated with executive function, and inhibitory ability was significantly associated with fundamental motor skills in boys; whereas locomotor skills showed significant associations with executive function, and both inhibitory ability and regulatory ability demonstrated significant associations with fundamental motor skills in girls.

### 4.1 The relationship between fundamental motor skills and executive function in children

This study identified a significant association between fundamental motor skills and executive function among Chinese children aged 3 to 10 years, a finding consistent with association patterns reported in existing literature (Diamond, 2000; Stöckel and Hughes, 2016). The direction of this motor-cognitive relationship aligns with prior evidence from preschool-aged populations (Cook et al., 2019; Yang et al., 2022; Vanhala et al., 2023; Jylänki et al., 2022; Gashaj et al., 2019; Han et al., 2022; Veldman et al., 2019; Albuquerque et al., 2022; Niederer et al., 2011; Piek et al., 2008; Niederer et al., 2011; Willoughby et al., 2021). Several studies have similarly documented association patterns between executive function and fundamental motor skills (Stuhr et al., 2020; Vanhala, 2024; Zelazo et al., 2016; Zelazo and Carlson, 2020; Mcclelland et al., 2013; Adolph and Hoch, 2019), a relationship implicating cognitive processes engaged during complex motor tasks, such as cognitive activities involved in creating and adapting motor plans according to task demands (Best, 2010). Current theoretical frameworks provide multiple explanatory perspectives for understanding this relationship: The reciprocal theory posits that motor and cognitive skills develop synergistically through environmental interactions, whereby movement experiences enhance motor competence, thereby improving environmental interaction efficacy, ultimately facilitating higher-order cognitive development (Kim et al., 2018; Maurer and Roebers, 2019). Furthermore, the automaticity theory proposes that complex skill acquisition depends on foundational skill automation. When tasks simultaneously demand motor and cognitive resources, these compete for limited attentional capacity. Automation of motor skills releases attentional resources to support cognitive task execution (Kim et al., 2018; Maurer and Roebers, 2019). Furthermore, neuroscientific evidence reveals shared neural substrates in the dorsolateral prefrontal cortex, cerebellum, and connecting structures underlying both fundamental motor skills and executive function (Diamond, 2000; Kim et al., 2018). Neuroimaging studies further delineate neural signatures of this motor-cognitive relationship, demonstrating cerebellar-prefrontal co-activation during complex tasks requiring rapid responses and focused attention, reflecting integrated motor coordination and executive processing (Diamond, 2000; Tomporowski et al., 2015). Consequently, the observation that children with higher fundamental motor skills exhibited lower executive function scores (indicating better executive performance, given the reverse scoring methodology of the executive function measure) demonstrates a statistically significant negative association. This reciprocal association pattern not only supports neurobiological evidence of motor-executive covariation during child development but also aligns with the aforementioned theoretical frameworks (reciprocal theory, automaticity theory, and neural foundations) (Diamond, 2000; Kim et al., 2018; Maurer and Roebers, 2019). This study further reveals distinctive bidirectional association patterns between fundamental motor skills and executive function in Chinese children aged 3–10 years, thereby expanding the evidentiary base in this field. Whereas traditional research often emphasizes unidirectional relationship hypotheses (e.g., motor-to-cognition or cognition-to-motor exclusively), our cross-sectional data demonstrate a significant, reciprocal statistical association. This reflects the statistical covariation between motor and cognitive capacities during early developmental stages. Given methodological variations in measurement tools across studies, we maintain cautious interpretation when comparing findings with related research.

### 4.2 Age-stratified association patterns between fundamental motor skills and executive function in children

A second key finding was the significant association patterns between executive function and fundamental motor skills across both age groups. Previous studies examining different developmental stages (Davis et al., 2011; Stuhr et al., 2020; Stöckel et al., 2017) have documented covariation between executive function and fundamental motor skills. Our findings extend this evidence by demonstrating age-dependent variation: stronger associations emerged in the 3-5-year-old group compared to the 6-10-year-old group, highlighting the moderating role of age in these relationship patterns.

Henri Wallon’s theoretical framework (Wallon, 1972; Shen and Wang, 2012) posits that children aged 3–5 years traverse a subjective period characterized by psychological transitions: diminishing role-play, strengthening self-assertion, active pursuit of competence validation, and eventual internalization of others’ strengths. Conversely, children aged 6–11 years enter an objective period where psychological focus shifts toward constructing systematic cognitive networks about the external world through synergistic social expansion and cognitive development. This perspective converges with Gallahue’s hourglass model of motor development (Gallahue and Donnelly, 2007; Salehi et al., 2017), which identifies ages 2–7 years as the peak environmental sensitivity period - a phase where fundamental motor skills (locomotion, manipulation, stability) achieve maximal neural plasticity through environmental input. Gabbard’s developmental continuum model further supports this view (Gabbard, 2021; Goodway et al., 2019), designating ages 2–7 years as the fundamental movement phase emphasizing critical pattern establishment, followed by the context-specific skill phase (7 years through adolescence) where foundational movements transform into specialized abilities. Subcomponent analyses revealed: Locomotor skills demonstrated associations with executive function in 3-5-year-olds; Object control skills showed associations with executive function in 6-10-year-olds Noting that boys aged 5–6 years typically outperform girls in object control assessments (Ke et al., 2020; Zheng et al., 2022; Honrubia-Montesinos and Losada-Puente, 2021). Executive function subcomponent analyses indicated: Inhibitory ability associated with fundamental motor skills in 3-5-year-olds; Working memory associated with fundamental motor skills in 6-10-year-olds. This finding aligns with Libertus’ proposition of age-dependent executive subcomponent specialization (Libertus and Hauf, 2017) and supports documented covariation patterns between motor competence and both inhibitory control and working memory in 5-6-year-olds within cross-sectional studies (Stöckel and Hughes, 2016). Piaget’s cognitive development theory (Pakpahan and Saragih, 2022) further elucidates this differentiation: the preoperational stage (3–7 years), characterized by egocentrism, features pronounced inhibitory control development, while the concrete operational stage (7–11 years), marked by logical thinking emergence, exhibits progressive working memory maturation.

Results demonstrated inhibitory ability associations with fundamental motor skills in 3-5-year-olds but not in 6-10-year-olds, whereas working memory associations emerged in the older group. This dissociation reflects heterogeneous developmental trajectories of executive subcomponents (Diamond, 2013; Best and Miller, 2010), wherein working memory shows progressive maturation during 6–10 years while inhibitory control develops most substantially during 3–5 years (Best and Miller, 2010). Supporting evidence comes from Koutsandréou et al. (2016) observation of working memory changes following 10-week motor interventions in 9-10-year-olds. These age-specific patterns likely represent distinct cognitive-motor integration signatures across developmental periods (Stuhr et al., 2020). Consistent with cognitive development frameworks (Best et al., 2009), inhibitory-motor associations predominate during 3–5 years while working memory-motor associations become prominent during 6–10 years. The persistence of working memory associations through preadolescence (Van Fels et al., 2019; Rigoli et al., 2012; Ludyga et al., 2018), aligns with its developmental trajectory and synergistic relationships with other executive components (Best and Miller, 2010; Hartung et al., 2020). When working memory reaches advanced developmental stages, other executive subcomponents may exhibit differential association patterns (Best et al., 2009). Our results exemplify this pattern through significant inhibitory-motor associations in early childhood (3–5 years) and working memory-motor associations in middle childhood (6–10 years).

### 4.3 Sex-stratified association patterns between fundamental motor skills and executive function in children

The third principal finding established statistically significant associations between executive function and fundamental motor skills in children of both genders (Mileva-Seitz et al., 2015; Yang et al., 2022), albeit with distinct gender-specific patterns. These differential patterns potentially arise from complex interactions involving sociocultural factors, environmental characteristics, and physical activity levels (LeGear et al., 2012). Boys demonstrated stronger overall association magnitudes than girls, with differential subdomain linkage characteristics: object control skills exhibited primary associations with executive function and inhibitory control in boys, whereas locomotor skills manifested principal associations with executive function, inhibitory control, and regulatory capacity in girls. This observation aligns with prior research documenting stronger correlations between fundamental motor skills and inhibitory control in boys (Yang et al., 2022), alongside evidence indicating superior performance among preschool girls in balance and manual dexterity assessments, contrasted with boys aged 5–6 years excelling in object control skill evaluations (Escolano-Pérez et al., 2022; Hirata et al., 2018; Ke et al., 2020; Smits-Engelsman et al., 2023; Navarro-Patón et al., 2021a; Mecías-Calvo et al., 2021; Zheng et al., 2022; Honrubia-Montesinos and Losada-Puente, 2021).

Such gender-differentiated patterns likely originate from multilevel interactions. At the neurobiological level, accelerated myelination processes within male sensorimotor cortices (particularly during ages 6–10 years) may underpin enhanced efficiency in fundamental movement execution. However, comparatively weaker prefrontal-limbic connectivity may increase reliance on foundational inhibitory control mechanisms (Sun et al., 2025; Johnson and De Haan, 2015). Conversely, girls may employ neural compensatory mechanisms integrating multimodal cognitive strategies, such as utilizing verbal encoding to facilitate motor learning (Gallahue and Donnelly, 2007). Socioculturally, gendered motor play preferences (e.g., boys’ frequent selection of ball-based activities) (Wang, 2022) and potential disparities in pedagogical practices (e.g., teachers prioritizing throwing skill instruction for boys) (Salvatori and Cherubini, 2024) persistently reinforce bio-social interactive effects. These interactions may selectively shape neural linkages between object manipulation skills and working memory in boys, while fostering associations between locomotor skills and multifaceted executive functions (e.g., inhibition and regulation) in girls.

These outcomes reflect the engagement profile of executive function components—particularly inhibitory control and regulatory capacity—during complex motor tasks, specifically within movement planning, online adjustment, sustained attentional focus, and environmental adaptation processes (Diamond, 2000). Current evidence substantiates inhibitory control, working memory (in certain studies), and regulatory capacity as salient correlates of motor skill proficiency. From an applied perspective, designing developmentally tailored interventions incorporating structured motor activities (e.g., aerobic exercise, martial arts), computerized cognitive training (e.g., N-back paradigms), or artistic group activities (e.g., drama, music), combined with healthy lifestyle practices (e.g., positive mindset, sufficient sleep) (Diamond and Ling, 2016; Diamond, 2012), may more effectively foster synergistic development of brain function and motor-cognitive abilities across genders. Motor-cognitive interventions require age-specific design, Preschoolers (3–5 years) benefit from locomotor activities with inhibitory challenges (e.g., rule-switching games), leveraging peak plasticity. School-aged children (6–10 years) need object-control tasks integrating working memory (e.g., tactical sports). Boy’s object-control advantage warrants gender-differentiated strategies. These theory-informed approaches transform statistical associations into targeted neuroeducation applications through movement-based pedagogy.

### 4.4 Limitations and implications

Notwithstanding its foundation in large-scale sample data and systematic analysis of age- and gender-stratified associations between fundamental motor skills (FMS) and executive function (EF) in children aged 3–10 years, this study exhibits limitations requiring consideration. The statistically significant negative FMS-EF correlations, while meaningful, demonstrated modest effect magnitudes (r = 0.06–0.12) within Cohen’s small-effect range (r = 0.10), suggesting real-world practical associations may be limited without synergistic dual-domain interventions. Firstly, the cross-sectional design precludes causal inference regarding developmental sequencing or underlying mechanisms between motor competence and executive function, necessitating longitudinal approaches to clarify dynamic interrelationships. Secondly, exclusive reliance on parent-reported EF measures risks reporting bias; future studies should implement multi-method assessments incorporating behavioral tasks and teacher ratings to enhance ecological validity. Finally, regional sampling from Eastern China may constrain generalizability, warranting validation across diverse geographical and cultural contexts to elucidate potential sociocultural influences on these association patterns.

## 5 Conclusion

This large-scale cross-sectional study examined age- and gender-stratified associations between fundamental motor skills and executive function in Chinese children aged 3–10 years. Analyses revealed bidirectional negative correlations between these domains, with stronger associations observed in preschoolers (3–5 years) compared to school-aged children (6–10 years). Distinct sex-specific patterns emerged in overall association strength and subcomponent-level relationships.

These findings demonstrate age-related and gender-based variations in motor-cognitive developmental linkages during childhood. The research contributes empirical evidence to understanding synergistic motor-executive development, underscoring the significance of concurrent engagement during sensitive periods.

Stratified patterns suggest developmentally tailored motor-cognitive engagement strategies: Preschool interventions could integrate locomotor activities with inhibitory challenges, while school-aged programs might combine object-control training with working memory tasks, considering sex-specific motor profiles. Such approaches may support integrated development during sensitive windows.

## Data Availability

The raw data supporting the conclusions of this article will be made available by the authors, without undue reservation.
